# Exploring the association between circulating trace elements, metabolic risk factors, and the adherence to a Mediterranean diet among children and adolescents with obesity

**DOI:** 10.3389/fpubh.2022.1016819

**Published:** 2023-01-13

**Authors:** Álvaro González-Domínguez, Jesús Domínguez-Riscart, María Millán-Martínez, Alfonso María Lechuga-Sancho, Raúl González-Domínguez

**Affiliations:** ^1^Instituto de Investigación e Innovación Biomédica de Cádiz (INiBICA), Hospital Universitario Puerta del Mar, Universidad de Cádiz, Cádiz, Spain; ^2^Unidad de Endocrinología Pediátrica y Diabetes, Servicio de Pediatría, Hospital Universitario Puerta del Mar, Cádiz, Spain; ^3^Associate Unit CSIC-University of Huelva “Atmospheric Pollution”, Center for Research in Sustainable Chemistry–CIQSO, University of Huelva, Huelva, Spain; ^4^Department of Chemistry, Faculty of Experimental Sciences, University of Huelva, Huelva, Spain; ^5^Departamento Materno Infantil y Radiología, Facultad de Medicina, Universidad de Cádiz, Cádiz, Spain

**Keywords:** childhood obesity, trace elements, Mediterranean diet, KIDMED, multi-elemental analysis

## Abstract

Diet is one of the most important modifiable lifestyle factors for preventing and treating obesity. In this respect, the Mediterranean diet (MD) has proven to be a rich source of a myriad of micronutrients with positive repercussions on human health. Herein, we studied an observational cohort of children and adolescents with obesity (*N* = 26) to explore the association between circulating blood trace elements and the degree of MD adherence, as assessed through the KIDMED questionnaire. Participants with higher MD adherence showed better glycemic/insulinemic control and a healthier lipid profile, as well as raised plasma levels of selenium, zinc, cobalt, molybdenum, and arsenic, and increased erythroid content of selenium. Interestingly, we found that these MD-related mineral alterations were closely correlated with the characteristic metabolic complications behind childhood obesity, namely hyperglycemia, hyperinsulinemia, and dyslipidemia (*p* < 0.05, |r| > 0.35). These findings highlight the pivotal role that dietary trace elements may play in the pathogenesis of obesity and related disorders.

## 1. Introduction

Obesity is a condition characterized by an excessive accumulation of body fat, which may have significant negative repercussions on health and raise the risk of developing other chronic diseases. The main driver of obesity is an imbalance between total energy intake and expenditure within the framework of a complex interplay involving multiple players, including genetic, metabolic, environmental, and behavioral factors (1). In this context, diet is well-recognized to be one of the most important modifiable lifestyle factors in the prevention and treatment of obesity and related disorders. On the one hand, the over-consumption of calories is the most likely cause of the obesity epidemic, mainly because of the loss of traditional dietary patterns and the increasingly frequent intake of energy-dense foods (e.g., high-fat foods) and added sugar-containing foods (2). Moreover, the adherence to unhealthy diets has also been linked to deficiencies of multiple micronutrients, including vitamins, carotenoids, and trace elements, which in turn may contribute and aggravate the pathogenic events behind obesity (3). Trace elements are essential micronutrients that are primarily acquired through the diet and participate in a myriad of primary biological process, including the antioxidant defense, immune system, hormonal regulation, and many others. In particular, growing evidence points to essential and toxic metals as pivotal regulators of a myriad of biological processes underlying the development and progression of obesity and its comorbidities (4). For instance, it has been repeatedly reported that numerous trace elements (e.g., zinc, chromium, vanadium, molybdenum, cobalt) play central roles in the synthesis, storage, and action of insulin, and consequently influence carbohydrate and lipid metabolism (5). Their correct homeostasis, both in terms of potentially pro-oxidant species (e.g., iron, copper) and elements participating in the antioxidant defense (e.g., selenium, manganese), is also crucial to maintain an adequate redox status (6, 7). Furthermore, it is noteworthy that metals and the immune system are bidirectionally interrelated, being mineral deficiencies associated with impaired immune function, whereas the inflammatory response (e.g., secretion of cytokines) can modulate the metabolism and bioavailability of trace elements and other nutrients (8). On the other hand, the exposure to toxic heavy metals (e.g., cadmium, mercury, lead) has been demonstrated to disrupt the endocrine system and to induce chronic inflammation and oxidative stress (9).

A few authors have previously studied the influence of diet determinants on the levels of specific metals among healthy children (10, 11), but this remains unexplored in obese populations. Obesity is nowadays the most prevalent chronic disease among children and adolescents, which can be often accompanied by different comorbidities related to abnormal insulin-mediated glucose control (e.g., insulin resistance, impaired fasting glucose, impaired glucose tolerance) and dyslipidemia (12). As mentioned above, insulin, glucose, and lipid metabolisms are tightly regulated by essential elements and may be impaired by exposure to toxic heavy metals. Accordingly, investigating the interplay between dietary habits, the homeostasis of trace elements, and the characteristic metabolic complications concurring with obesity is crucial to get new insights into the pathogenesis of this disorder. In this study, we aimed to explore the association between circulating trace elements and the degree of adherence to a Mediterranean diet (MD), as assessed through the Mediterranean Diet Quality Index for children and adolescents (KIDMED), in a Spanish observational cohort of children and adolescents with obesity. To this end, we applied multi-elemental analysis to plasma and erythrocyte samples with the aim of comprehensively characterizing the biodistribution of trace elements in peripheral blood. Furthermore, we also investigated the association between blood metals and other biochemical variables (e.g., glycemia, insulinemia, lipid profile) to better understand the role of diet-related micronutrient alterations in the pathogenic events behind childhood obesity.

## 2. Materials and methods

### 2.1. Study design

The study design consisted of an observational cohort of children and adolescents with obesity recruited at “Hospital Universitario Puerta del Mar” (Cádiz, Spain), who underwent an oral glucose tolerance test (OGTT) under medical prescription. The inclusion criteria were children and adolescents of both sexes, aged between 6 and 14 years, presenting a body mass index (BMI) over two standard deviations above the mean of the reference population, adjusted for sex and age (13). Subjects with other known chronic systemic diseases or suffering of acute infectious processes were not eligible for the study. Using a sample size of 26 children and considering an alpha risk of 0.05, the statistical power of our comparisons was above 80%, as calculated using the GRANMO 7.12 webtool. The study was performed in accordance with the principles contained in the Declaration of Helsinki. The Ethical Committee of “Hospital Universitario Puerta del Mar” (Cádiz, Spain) approved the study protocol (Ref. PI22/01899), and all participants and/or legal guardians provided written informed consent.

### 2.2. Sampling and determination of anthropometric and biochemical variables

From all participants, venous blood samples were obtained by venipuncture of the antecubital region using an intravenous catheter, BD Vacutainer tubes, and Advance vacuum system. All baseline blood samples were collected in the morning after overnight fasting to minimize the influence of the circadian rhythm and dietary factors. Additional blood samples were collected at the different time points along the OGTT curve (i.e., 30, 60, 90, and 120 min) to evaluate glycemia- and insulinemia-related variables. After withdrawal, blood tubes were gently inverted several times and placed horizontally on ice to prevent red cell lysis and to reduce protease activity. Then, blood samples were centrifuged at 1,500 g for 10 min at 4°C to separate the plasma. Finally, the resulting pellets were washed with cold saline solution (9 g/L NaCl, 4°C) and centrifuged at 1,500 g for 5 min at 4°C (three times) to obtain the erythrocyte fraction. The plasma and erythrocyte samples were aliquoted and stored at −80°C until analysis.

Anthropometric variables (i.e., weight, height, BMI, and waist circumference) were evaluated by pediatric endocrinologists (J.D.-R. and A.M.L.-S.). The height was measured using a fixed wall stadiometer, whereas weight was determined in a SECA 5,000 balance. Blood glucose and insulin concentrations along the OGTT curve (i.e., 0, 30, 60, 90, and 120 min), as well as the fasting lipid profile, were measured using an Alinity automatic analyzer (Abbot, Spain) located at the Clinical Analysis Laboratory of the “Hospital Universitario Puerta del Mar.” Briefly, plasma levels of glucose and insulin were determined using the hexokinase method (CV <3%) and an electrochemiluminescence microparticle immunoassay (CV <2.2%), respectively. Enzymatic colorimetric assays were applied to quantify total cholesterol (TC, CV <1.4%), high-density lipoprotein cholesterol (HDL-C, CV <2.1%), low-density lipoprotein cholesterol (LDL-C, CV <1.7%), and triglycerides (TG, CV <1.1%). The homeostasis model assessment of insulin resistance (HOMA-IR) was calculated by applying the following formula: HOMA-IR = (Glc_0_ × Ins_0_) × 0.055/22.5, where Glc_0_ and Ins_0_ refer to the fasting plasma levels of glucose (mg/dL) and insulin (μU/mL), respectively.

### 2.3. Dietary assessment

The adherence to a MD of the study participants was assessed using the updated version of the Mediterranean Diet Quality Index for children and adolescents (KIDMED) (14, 15). This dietary questionnaire consists of 16 dichotomous items (i.e., yes/no), of which 12 questions denote a positive connotation with respect to the MD (e.g., consumption of fruits, vegetables, fish, pulses, pasta or rice, cereals, nuts, olive oil, cereals, dairy products) and 4 questions denote a negative connotation (e.g., consumption of fast food, baked goods, sweets, skipping breakfast). Items with a positive connotation were assigned a value of +1, and those with a negative connotation a value of −1. The total KIDMED score is obtained by summing these individual values, which allows classifying subjects into three different groups according to their degree of MD adherence: very low diet quality (≤3), improvement needed to adjust intake to Mediterranean patterns (4–7), and optimal MD (≥8). For further analyses, these categories were collapsed into low MD adherence (“improvement needed to adjust intake to Mediterranean patterns” and “very low diet quality”) or high MD adherence (“optimal MD”) (16). This dietary assessment was conducted *via* interview by pediatric endocrinologists (J.D.-R.).

### 2.4. Multi-elemental analysis of plasma and erythrocyte samples

Trace elements (i.e., vanadium, chromium, manganese, iron, cobalt, copper, zinc, arsenic, selenium, molybdenum, cadmium, and lead) were determined by diluting aliquots of 150 μL of plasma or 50 μL of the erythrocyte fraction to a final volume of 3 mL using an alkaline solution containing 2% 1-butanol (w/v), 0.05% EDTA (w/v), 0.05% Triton X-100 (w/v), and 1% NH_4_OH (w/v) (17). As the internal standard, rhodium was added to sample extracts to reach a final concentration of 1 μg/L. Samples were filtered through 0.45 μm pore size hydrophilic PTFE filters before analysis. Then, multi-elemental determinations were performed using an Agilent 7,900 inductively coupled plasma mass spectrometer (ICP-MS) equipped with collision/reaction cell system and with nickel sampling and skimmer cones (Agilent Technologies, Tokyo, Japan). High-purity grade helium (>99.999%) was employed as the collision gas. The ICP-MS working conditions were set as follows (18): sampling depth, 7 mm; forward power, 1,550 W; plasma gas, 15 L/min; auxiliary gas, 1 L/min; carrier gas, 1 L/min; make-up gas, 0.10 L/min; collision gas, 5 mL/min. The isotopes monitored were ^51^V, ^52^Cr, ^53^Cr, ^55^Mn, ^56^Fe, ^57^Fe, ^59^Co, ^63^Cu, ^66^Zn, ^75^As, ^77^Se, ^78^Se, ^82^Se, ^95^Mo, ^98^Mo, ^103^Rh, ^111^Cd, and ^208^Pb, using a dwell time of 0.3 s per isotope. Multi-elemental calibration curves were prepared within the concentration range 0.5–2,500 μg/L, containing 1 μg/L rhodium as the internal standard.

### 2.5. Statistical analysis

Data normality and skewness was first tested by inspecting normal probability plots and by performing the Kolmogorov–Smirnov test. Clinical, biochemical, and dietary variables were subjected to Mann-Whitney U test (continuous variables) and chi-square test (dichotomous variables) to compare the study groups. The pre-processing and statistical analysis of the multi-elemental data were performed using the MetaboAnalyst 5.0 web tool (https://www.metaboanalyst.ca/), as follows. First, variables with more than 20% missing values were removed, and the remaining missing values were imputed using the kNN algorithm. Then, the data were log transformed and Pareto scaled. To look for differences between the study groups, data were subjected to Mann-Whitney U test. Finally, correlation analysis was applied to look for associations between trace elements and the KIDMED scores, as well as between trace elements and other biochemical variables (i.e., glucose, insulin, lipids). *P*-Values below 0.05 were considered as statistically significant.

## 3. Results

In our study population of children and adolescents with obesity, the demographic and anthropometric characteristics were similar between participants with low and high MD adherence as assessed through the KIDMED questionnaire (Table 1). Evidently, the average KIDMED scores significantly differed between the study groups (low MD adherence: 5.2, high MD adherence: 8.3), and similar results were observed for the frequency of consumption of some of the individual items conforming the questionnaire (e.g., Q2: consumption of fruits; Q9: consumption of cereals). Furthermore, it should be noted that subjects reporting higher KIDMED scores showed lower blood levels of glucose and insulin along the OGTT curve, as well as lower fasting levels of total cholesterol.

**Table 1:** Demographic, anthropometric, dietary, and biochemical characterization of the study population.

	**Low MD adherence**	**High MD adherence**	***p*-value**
**Demographics and anthropometry**			
*N*	12	14	
Age (years)	10.4 [9.3–11.8]	11.7 [10.3–13.0]	NS
Sex (% male)	56.7	57.1	NS
Weight (kg)	68.8 [58.0–80.2]	70.8 [67.8–80.4]	NS
Body mass index (kg/m^2^)	31.1 [28.8–35.2]	30.5 [27.7–32.0]	NS
Waist circumference (cm)	103.5 [95.0–113.0]	102.0 [98.0–105.5]	NS
**Dietary assessment**			
KIDMED score	5.5 [4.0–6.0]	8.0 [7.0–9.0]	1.6·10^−7^
Q1. Consumes a fruit every day (%)	58.3	85.7	NS
Q2. Consumes a second fruit every day (%)	25.0	71.4	1.8·10^−2^
Q3. Consumes fresh/cooked vegetables regularly once a day (%)	41.7	64.3	NS
Q4. Consumes fresh/cooked vegetables more than once a day (%)	16.7	28.6	NS
Q5. Consumes fish regularly (%)	58.3	78.5	NS
Q6. Consumes fast-food more than once a week (%)	25.0	7.1	NS
Q7. Consumes pulses more than once a week (%)	83.3	100.0	NS
Q8. Consumes pasta/rice almost every day (%)	8.3	0.0	NS
Q9. Consumes cereals/grains for breakfast (%)	41.7	78.6	4.9·10^−2^
Q10. Consumes nuts regularly (%)	25.0	42.9	NS
Q11. Consumes olive oil at home (%)	100.0	100.0	NS
Q12. Skips breakfast (%)	25.0	14.3	NS
Q13. Consumes a dairy product for breakfast (%)	66.7	92.9	NS
Q14. Consumes commercially baked goods or pastries for breakfast (%)	25.0	14.3	NS
Q15. Consumes two yogurts and/or some cheese daily (%)	66.7	78.6	NS
Q16. Consumes sweets and candy several times every day (%)	16.7	14.3	NS
**Biochemical variables**			
Glucose, t = 0' (Glc_0_, mg/dL)	81.0 [76.2–84.2]	83.5 [78.0–86.0]	NS
Glucose, t = 30' (Glc_30_, mg/dL)	139.0 [122.2–157.2]	143.0 [130.0–159.0]	NS
Glucose, t = 60' (Glc_60_, mg/dL)	143.0 [126.5–161.2]	120.0 [112.0–138.8]	3.8·10^−2^
Glucose, t = 90' (Glc_90_, mg/dL)	128.0 [118.8–148.5]	108.5 [100.0–115.8]	2.6·10^−3^
Glucose, t = 120' (Glc_120_, mg/dL)	134.0 [124.0–144.5]	124.5 [115.0–127.8]	2.6·10^−2^
Insulin, t = 0' (Ins_0_, μU/mL)	17.0 [14.0–21.5]	16.5 [13.0–20.8]	NS
Insulin, t = 30' (Ins_30_, μU/mL)	125.1 [84.4–156.5]	132.6 [77.0–185.7]	NS
Insulin, t = 60' (Ins_60_, μU/mL)	135.7 [96.0–226.6]	143.4 [126.5–162.2]	NS
Insulin, t = 90' (Ins_90_, μU/mL)	127.4 [96.6–159.7]	109.3 [74.0–141.8]	NS
Insulin, t = 120' (Ins_120_, μU/mL)	147.1 [123.4–280.2]	107.9 [106.5–170.4]	2.2·10^−2^
Homeostasis model assessment of insulin resistance (HOMA-IR)	3.3 [2.8–3.9]	3.2 [2.6–3.8]	NS
Total Cholesterol (TC, mg/dL)	159.5 [149.8–166.2]	149.5 [119.0–152.0]	4.0·10^−3^
High-density lipoprotein cholesterol (HDL-C, mg/dL)	43.5 [40.0–49.8]	40.0 [38.0–45.8]	NS
Low-density lipoprotein cholesterol (LDL-C, mg/dL)	96.5 [93.2–102.5]	96.5 [70.0–98.0]	NS
Triglycerides (TG, mg/dL)	86.0 [60.0–103.5]	92.0 [82.0–117.5]	NS

Results are expressed as median [IQR] (except for dichotomous variables, expressed as percentages). NS, non-significant.

**Figure 1:**
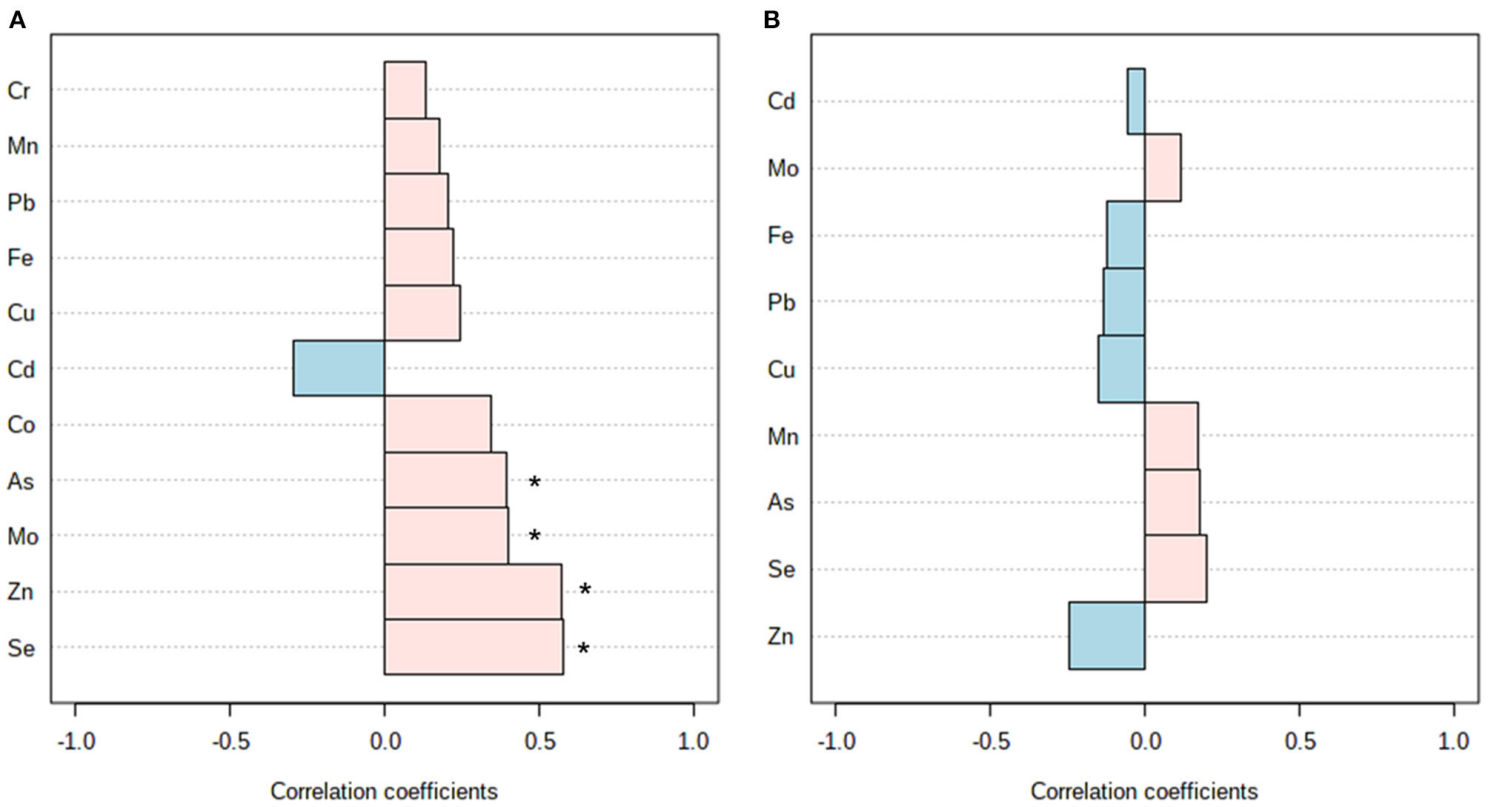
Correlation analysis between the KIDMED score and multi-elemental data from plasma **(A)** and erythrocytes **(B)**. *Denotes significant correlation (*p* < 0.05).

The degree of adherence to a MD also had a significant influence on the multi-elemental profile of plasma and erythrocyte samples of the study participants, as shown in Table 2. The plasma contents of selenium, zinc, cobalt, molybdenum, and arsenic were higher among subjects adhering to an optimal MD, and the same trend was observed for other trace elements without reaching statistical significance (e.g., chromium, manganese, iron). Similarly, increased erythroid selenium was observed in children and adolescents from the high MD adherence group, but no significant differences were found for the rest of elements under investigation. These findings were further corroborated in a large extent through correlation analyses. The KIDMED index was found to be positively associated with plasma levels of selenium (r = 0.58), zinc (r = 0.57), molybdenum (r = 0.40), and arsenic (r = 0.39) (Figure 1A). Furthermore, circulating trace elements were also correlated with the frequency of consumption of some of the individual items conforming the KIDMED questionnaire (Supplementary Figure 1). A consistent negative association was observed between unhealthy dietary habits (e.g., consumption of fast food, baked goods, sweets) and the plasma contents of zinc and/or molybdenum. On the other hand, the intake of food products with a healthy connotation with respect to the MD was positively associated with a myriad of plasmatic micronutrients: the consumption of a second fruit with molybdenum and iron, fish with selenium and arsenic, pulses with molybdenum, and dairy products with copper. Conversely, correlation analysis between dietary intake data and erythroid mineral profiles yielded less robust results, with only a few individual food items being significantly associated with copper (e.g., consumption of a second vegetable, cereals) and zinc (e.g., consumption of pasta/rice) levels (Supplementary Figure 2).

**Table 2:** Plasma and erythroid concentrations of trace elements in the two study groups.

	**Plasma**			**Erythrocytes**		

	**Low MD adherence**	**High MD adherence**	***p*** **-value**	**Low MD adherence**	**High MD adherence**	***p*** **-value**
Chromium	3.4 [2.9–4.9]	4.8 [3.7–7.5]	NS	ND	ND	-
Manganese	4.0 [3.7–4.5]	4.7 [3.6–5.0]	NS	20.2 [10.9–27.7]	18.7 [14.2–28.0]	NS
Iron	676.8 [622.8–723.7]	779.0 [659.8–835.4]	NS	5.8·10^5^ [5.6·10^5^-6.1·10^5^]	5.6·10^5^ [5.4·10^5^-5.8·10^5^]	NS
Cobalt	0.57 [0.53–0.62]	0.68 [0.61–0.73]	2.2·10^−2^	ND	ND	-
Copper	1,207.6 [1,122.8–1,311.0]	1,228.9 [1,171.2–1,301.8]	NS	640.5 [590.6–666.6]	631.9 [584.7–687.7]	NS
Zinc	689.2 [566.7–1,176.0]	1,215.5 [1,156.3–1,273.5]	9.5·10^−3^	10,047.3 [9,479.1–10,787.2]	9,547.5 [9,184.2–9,753.2]	NS
Arsenic	0.57 [0.33–0.90]	0.85 [0.71–1.09]	2.8·10^−2^	42.0 [31.2–50.1]	47.0 [40.0–50.9]	NS
Selenium	124.4 [120.7–133.5]	137.6 [131.5–143.9]	3.3·10^−3^	164.4 [136.1–186.1]	179.5 [159.6–242.2]	3.7·10^−2^
Molybdenum	1.9 [1.4–2.4]	2.7 [2.3–3.1]	2.8·10^−2^	19.3 [17.2–22.7]	21.1 [19.9–24.8]	NS
Cadmium	0.0026 [0.00074–0.0036]	0.0018 [0.00099–0.0027]	NS	1.9 [1.3–2.6]	1.8 [1.1–2.6]	NS
Lead	0.021 [0.020–0.022]	0.021 [0.020–0.023]	NS	62.4 [58.3–71.3]	58.4 [56.5–60.2]	NS

Results are expressed as median [IQR] (μg/L). ND, non-detected; NS, non-significant.

**Figure 2:**
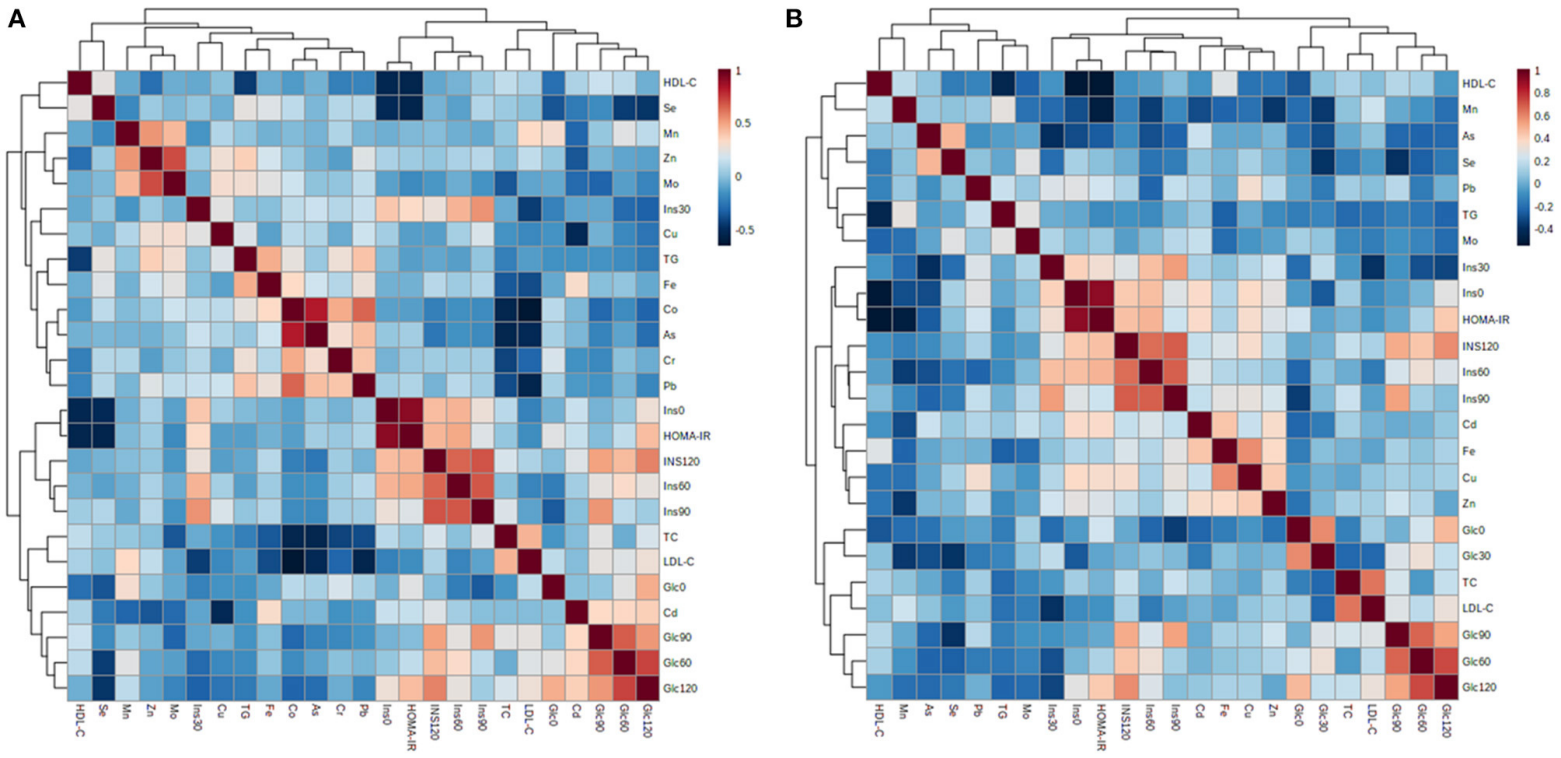
Pearson's correlation matrix between biochemical variables and multi-elemental data from plasma **(A)** and erythrocytes **(B)**.

Finally, Pearson's correlations were computed between multi-elemental and biochemical variables with the aim of investigating the relationship between diet-related micronutrient alterations and the typical metabolic complications behind childhood obesity (Figure 2). Glucose and insulin concentrations along the OGTT, as well as the HOMA-IR scores, showed a consistent negative association with selenium (r = −0.36 with Glc_0_, r = −0.43 with Glc_60_, r = −0.45 with Glc_120_, r = −0.50 with Ins_0_, r = −0.54 with HOMA-IR) and a positive association with cadmium (r = 0.35 with Glc_60_, r = 0.35 with Glc_90_, r = 0.38 with Glc_120_) in plasma. The same trend of association was observed for erythroid selenium (r = −0.37 with Glc_30_, r = −0.38 with Glc_90_) and cadmium (r = 0.37 with Ins_0_, r = 0.37 with HOMA-IR). Similarly, erythroid levels of manganese were negatively correlated to the HOMA-IR score (r = −0.48). Regarding lipid parameters, various plasmatic trace elements, including iron, cobalt, chromium, arsenic, and lead, were negatively associated with LDL-C (r = −0.38 with Fe, r = −0.63 with Co, r = −0.51 with As, r = −0.54 with Pb) and/or TC (r = −0.34 with Fe, r = −0.53 with Co, r = −0.40 with Cr, r = −0.53 with As, r = −0.38 with Pb) levels. Conversely, a positive association was found between TGs and plasma iron (r = 0.48), zinc (r = 0.38), and lead (r = 0.42).

## 4. Discussion

Dietary assessment through self-reporting methods (e.g., food frequency questionnaires, dietary recalls) has repeatedly evidenced a positive association between the adherence to a MD, micronutrient intake, and nutritional adequacy in young (19, 20), adult (21), and older populations (22). However, the measurement of dietary biomarkers in biological matrices has emerged in recent years as a more reliable strategy to get a closer and more objective understanding of the crosstalk between diet and health within the complex meshwork of bioavailability, metabolism, biodistribution, and excretion processes. In this respect, some observational studies have previously reported that subjects adhering to a MD usually have increased blood levels of multiple micronutrients, including vitamins (23), lipid-soluble compounds (e.g., carotenoids, tocopherols) (24), n-3 polyunsaturated fatty acids (25), and other essential food bioactives. To get a more comprehensive exploration about the influence of nutrition on human health, other authors have proposed the application of wide-coverage metabolomics approaches for identifying food intake biomarkers and biomarkers of adherence to specific dietary patterns (26, 27). Nevertheless, the number of studies relying on the determination of trace elements are still scarce (28), and normally focused on investigating single food items rather than complex dietary patterns (10, 11, 29).

In the present study, we have demonstrated for the first time that circulating blood trace elements can reflect the degree of MD adherence, as assessed through the KIDMED index, among children and adolescents with obesity. Herein, we found that subjects reporting higher KIDMED scores showed increased selenium, zinc, cobalt, molybdenum, and arsenic in plasma, as well as increased erythroid levels of selenium (Table 2). These findings could be explained by the rich content of minerals present in the major food products that conform the traditional MD, including vegetables, fruits, cereals, and fish (30). Indeed, nutritional interventions with heathy dietary habits (e.g., MD) have proven to significantly raise the serum concentrations of various essential minerals (31). Further correlation analyses corroborated our results (Figure 1) and shed more light on the specific food items that could contribute the most to the characteristic multi-elemental profiles observed among participants with optimal MD adherence (Supplementary Figures 1, 2). Circulating contents of essential trace elements (e.g., selenium, iron, molybdenum, copper) were positively correlated with the frequency of consumption of healthy food products, such as fruits, vegetables, fish, pulses, and dairy products, in line with previous data (29, 32). Conversely, plasma levels of zinc and molybdenum were negatively associated with unhealthy dietary habits, such as the intake of fast foods, baked goods, and sweets. Therefore, we hypothesize that metal differences between the study groups could be regarded as a direct reflection of the MD adherence, since participants consuming heathy diets are expected to have higher circulating levels of minerals than subjects who replace healthy food items with micronutrient-poor ones. Altogether, these results highlight the crucial role that diet may play on maintaining an adequate micronutrient status. Nevertheless, it is worth mentioning here that levels of metal elements may be influenced by many exogenous (e.g., diet, pollution, smoking, medications) and endogenous factors (e.g., sex, age, hormonal factors) (33), which considerably limits their reliability as sensitive and specific dietary biomarkers.

To conclude, it should also be noted that participants with the higher MD adherence showed better glycemic and insulinemic control (i.e., lower blood levels of glucose and insulin along the OGTT curve) and a healthier lipid profile (i.e., lower fasting TC levels) compared to those reporting lower KIDMED scores. For this reason, we decided to explore possible associations between the MD-related mineral profile and the pathogenic hallmarks behind childhood obesity, namely abnormal insulin-mediated carbohydrate metabolism and dyslipidemia (Figure 2). On the one hand, we found a strong negative association between selenium and manganese levels (this latter only in erythrocytes) and different variables related to hyperglycemia and hyperinsulinemia. In this respect, other authors have previously reported reduced activity of selenium- and manganese-dependent antioxidant enzymes, such as glutathione peroxidase (34, 35) and manganese superoxide dismutase (36), in children with obesity. This exacerbated oxidative stress may in turn perturb insulin secretion in pancreatic β cells (5), which could explain the direction of association between the above-mentioned trace elements and glycemia/insulinemia-related variables. Conversely, the association was positive with plasma and erythroid contents of cadmium, plausibly because of its capacity to disrupt the endocrine system, provoke insulin resistance, and consequently increase the circulating concentrations of insulin and glucose (9). In line with previous studies describing the involvement of trace elements and heavy metals in the development of dyslipidemia factors, we found a negative correlation between various blood cholesterol fractions (e.g., TC, LDL-C) and plasmatic minerals (e.g., iron, cobalt, chromium, arsenic, lead). This could be attributed to the essential roles that trace elements seem to play in ameliorating atherogenic dyslipidemia through a myriad of mechanisms (e.g., lipid β-oxidation, expression of peroxisome proliferator-activated receptors), and particularly by interfering with cholesterol metabolism (37–39). Surprisingly, a positive association was found between plasma TG, iron, zinc, and lead, which could be due to their dietary co-occurrence (e.g., animal-based products).

## 5. Conclusions

Herein, we have demonstrated that the degree of MD adherence, as assessed through the KIDMED score, has a significant impact on the biochemical profile and trace element status among children and adolescents with obesity. Subjects adhering to an optimal MD showed better glycemic/insulinemic control and a healthier lipid profile, as well as increased metal contents in plasma (selenium, zinc, cobalt, molybdenum, arsenic) and erythrocytes (selenium). Further correlation analyses evidenced close interrelationships between these diet-related mineral alterations and the typical metabolic complications behind childhood obesity (i.e., abnormal insulin-mediated carbohydrate metabolism, dyslipidemia), thereby highlighting the crucial role that diet might play on health through maintaining an adequate micronutrient status.

The main limitation of the present study was the relatively small sample size of the population under investigation and the lack of an independent cohort for validation purposes. Although this pilot exploration lays the foundation for better understanding the interplay between dietary habits, homeostasis of trace elements, and obesity-related pathogenic events, future studies are needed in larger cohorts to further validate our findings. In this respect, the analysis of other complementary biological matrices could be of great interest to investigate short-term (e.g., urine) and long-term (e.g., hair, nails) exposure.

## Data availability statement

The raw data supporting the conclusions of this article will be made available by the authors, without undue reservation.

## Ethics statement

The studies involving human participants were reviewed and approved by Ethical Committee of “Hospital Universitario Puerta del Mar” (Cádiz, Spain). Written informed consent to participate in this study was provided by the participants' legal guardian/next of kin.

## Author contributions

RG-D: conceptualization, project administration, and supervision. ÁG-D and RG-D: data curation and roles/writing–original draft. ÁG-D, MM-M, and RG-D: formal analysis. RG-D and AL-S: funding acquisition and resources. ÁG-D, MM-M, JD-R, AL-S, and RG-D: investigation and writing–review and editing. MM-M and RG-D: methodology. All authors have read and agreed to the published version of the manuscript.
