# Supplementary material for: Exploring the association between circulating trace elements, metabolic risk factors, and the adherence to a Mediterranean diet among children and adolescents with obesity
Source: Front Public Health. 2023 Jan 13;10:1016819. doi: 10.3389/fpubh.2022.1016819 (PMC9880061; doi:10.3389/fpubh.2022.1016819)
Supplement: Supplementary file 1 [file Data_Sheet_1.docx]

Supplementary Material


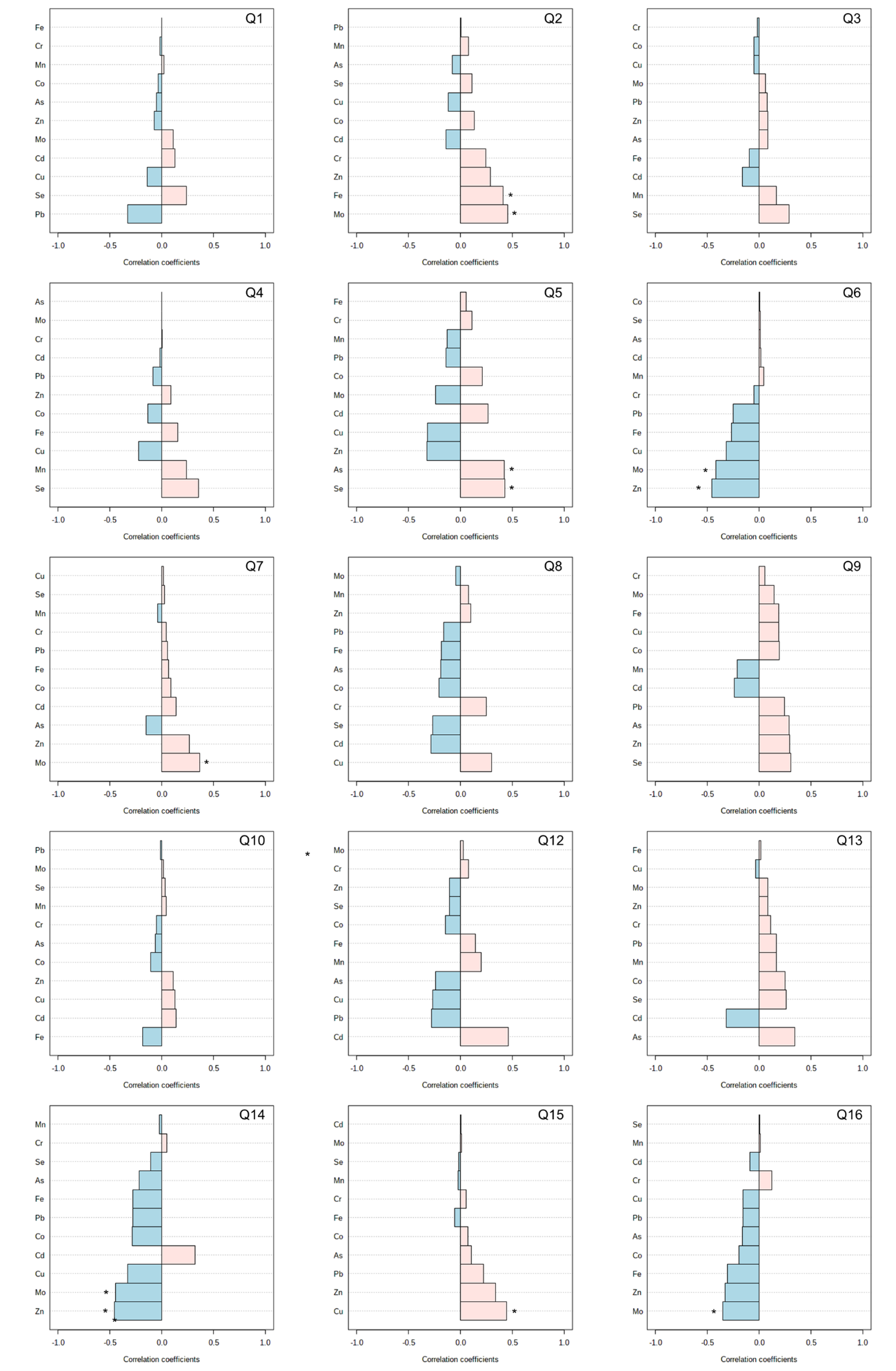


**Supplementary Figure 1.** Correlation analysis between the individual KIDMED items (Q1-Q16) and plasma multi-elemental data. Q1: consumes a fruit every day, Q2: consumes a second fruit every day, Q3: consumes fresh/cooked vegetables regularly once a day, Q4: consumes fresh/cooked vegetables more than once a day, Q5: consumes fish regularly Q6: consumes fast-food more than once a week, Q7: consumes pulses more than once a week, Q8: consumes pasta/rice almost every day, Q9: consumes cereals/grains for breakfast, Q10: consumes nuts regularly, Q12: skips breakfast, Q13: consumes a dairy product for breakfast, Q14: consumes commercially baked goods or pastries for breakfast, Q15: consumes two yoghurts and/or some cheese daily, Q16: consumes sweets and candy several times every day. *Denotes significant correlation (p-value < 0.05).


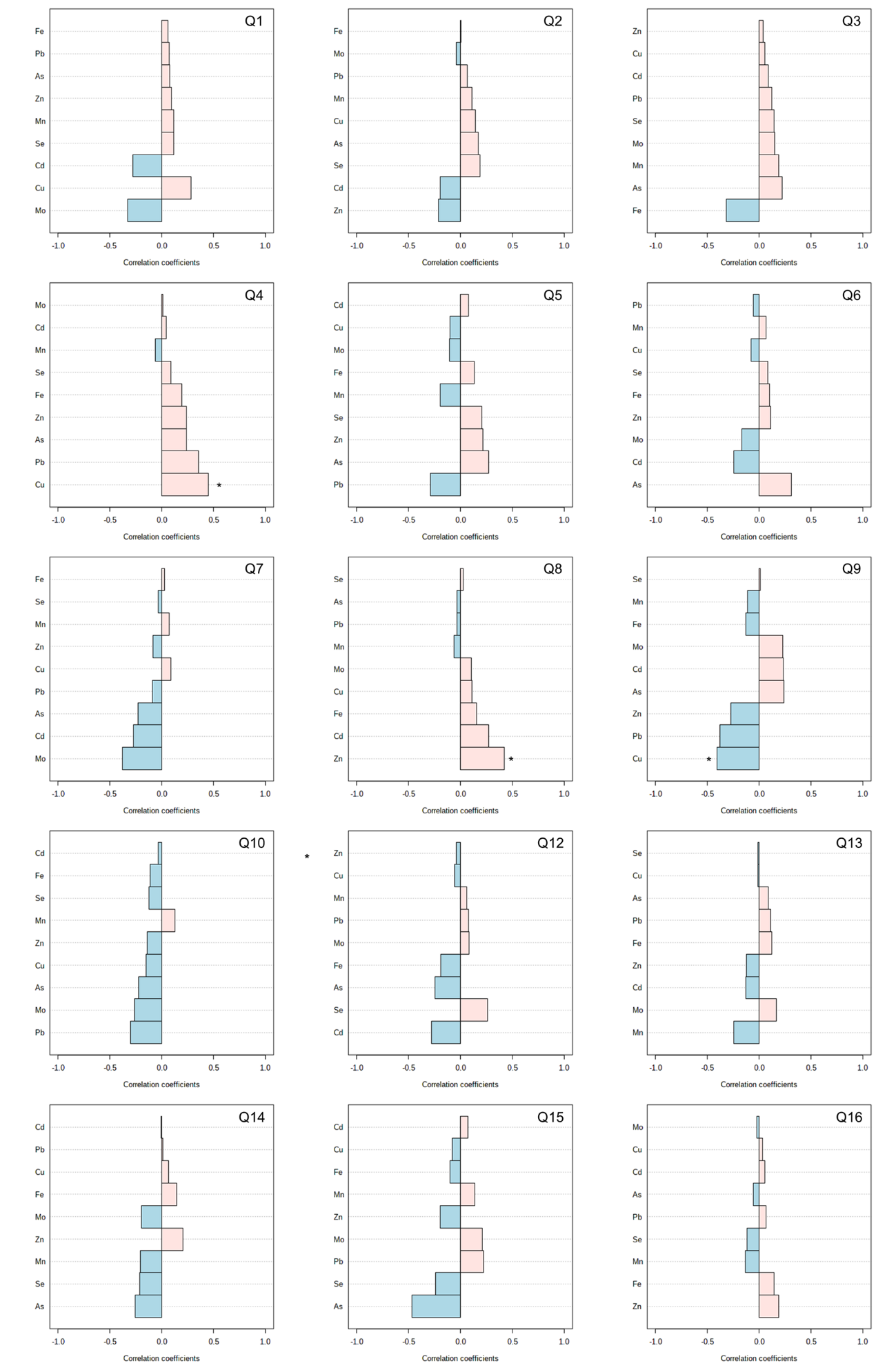


**Supplementary Figure 2.** Correlation analysis between the individual KIDMED items (Q1-Q16) and erythroid multi-elemental data. Q1: consumes a fruit every day, Q2: consumes a second fruit every day, Q3: consumes fresh/cooked vegetables regularly once a day, Q4: consumes fresh/cooked vegetables more than once a day, Q5: consumes fish regularly Q6: consumes fast-food more than once a week, Q7: consumes pulses more than once a week, Q8: consumes pasta/rice almost every day, Q9: consumes cereals/grains for breakfast, Q10: consumes nuts regularly, Q12: skips breakfast, Q13: consumes a dairy product for breakfast, Q14: consumes commercially baked goods or pastries for breakfast, Q15: consumes two yoghurts and/or some cheese daily, Q16: consumes sweets and candy several times every day. *Denotes significant correlation (p-value < 0.05).
